# Development of a Novel Surfactant-Based Viscoelastic Fluid System as an Alternative Nonpolymeric Fracturing Fluid and Comparative Analysis with Traditional Guar Gum Gel Fluid

**DOI:** 10.3390/polym15112444

**Published:** 2023-05-25

**Authors:** Mahesh Chandra Patel, Mohammed Abdalla Ayoub, Mazlin Bt Idress, Anirbid Sircar

**Affiliations:** 1 Department of Petroleum Engineering, Universiti Teknologi Petronas, Perak 32610, Malaysia; 2 School of Petroleum Technology, Pandit Deendayal Energy University, Gandhinagar 382007, Gujarat, India

**Keywords:** surfactant-based viscoelastic fluids for fracturing, viscoelastic surfactant fluids, ZnO nanoparticle-assisted viscoelastic fluids, alternative nonpolymeric fracturing fluid compositions, CTAB-based viscoelastic fluids, rheology of guar gum, comparison of polymeric and nonpolymeric viscoelastic fluids

## Abstract

Surfactant-based viscoelastic (SBVE) fluids have recently gained interest from many oil industry researchers due to their polymer-like viscoelastic behaviour and ability to mitigate problems of polymeric fluids by replacing them during various operations. This study investigates an alternative SBVE fluid system for hydraulic fracturing with comparable rheological characteristics to conventional polymeric guar gum fluid. In this study, low and high surfactant concentration SBVE fluid and nanofluid systems were synthesized, optimized, and compared. Cetyltrimethylammonium bromide and counterion inorganic sodium nitrate salt, with and without 1 wt% ZnO nano-dispersion additives, were used; these are entangled wormlike micellar solutions of cationic surfactant. The fluids were divided into the categories of type 1, type 2, type 3, and type 4, and were optimized by comparing the rheological characteristics of different concentration fluids in each category at 25 °C. The authors have reported recently that ZnO NPs can improve the rheological characteristics of fluids with a low surfactant concentration of 0.1 M cetyltrimethylammonium bromide by proposing fluids and nanofluids of type 1 and type 2. In addition, conventional polymeric guar gum gel fluid is prepared in this study and analyzed for its rheological characteristics. The rheology of all SBVE fluids and the guar gum fluid was analyzed using a rotational rheometer at varying shear rate conditions from 0.1 to 500 s^−1^ under 25 °C, 35 °C, 45 °C, 55 °C, 65 °C, and 75 °C temperature conditions. The comparative analysis section compares the rheology of the optimal SBVE fluids and nanofluids in each category to the rheology of polymeric guar gum fluid for the entire range of shear rates and temperature conditions. The type 3 optimum fluid with high surfactant concentration of 0.2 M cetyltrimethylammonium bromide and 1.2 M sodium nitrate was the best of all the optimum fluids and nanofluids. This fluid shows comparative rheology to guar gum fluid even at elevated shear rate and temperature conditions. The comparison of average viscosity values under a different group of shear rate conditions suggests that the overall optimum SBVE fluid prepared in this study is a potential nonpolymeric viscoelastic fluid candidate for hydraulic fracturing operation that could replace polymeric guar gum fluids.

## 1. Introduction

Fracturing is a widely used technique that involves pumping highly pressurized fluid to break up hydrocarbon-bearing formations, then pumping a proppant slurry (a thickening agent carrying proppant) into these artificially created fractures to keep them open, allowing hydrocarbon (oil or gas) production rate from the well to be significantly improved [1,2]. The viscosity of fracturing fluid is an important and key parameter during both the fracture creation and proppant transport phases of fracturing operations. It is important to consider the factors which can lead to degradation of fractures, such as the non-Newtonian nature of fracture fluid and the increase in viscosity of proppant slurry due to proppant concentration during consideration of the optimal fluid viscosity. The fracturing fluid provides perfect proppant transport if it retains viscosity of 50 to 100 cp under reservoir temperatures and 170 s^−1^ shear rate conditions [3]. The industry accepts a minimum guideline for a fracturing fluid of viscosity property 100 cp at a 100 s^−1^ shear rate for adequate proppant transport and placement [4]. Recently, Gaurina-Međimurec et. al. (2021) reported that the fluid viscosity of fracturing fluid should be high enough (from 100 to 1000 cp) to create the fracture and to enable proppant entrance into the fracture [5].

**Figure 1:**
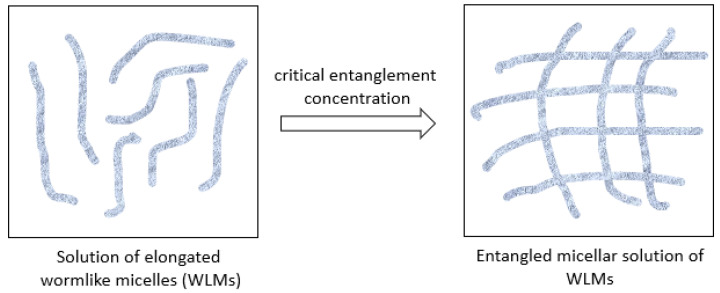
Formation of SBVE fluid system of entangled micellar solution of WLMs.

Surfactant-based viscoelastic fluids have recently been used in hydraulic fracturing operations, where they have begun to replace fracking fluids based on polymers [2,6]. Polymer fluids such as guar gum gels are the main compositions used as fracturing fluid thickeners during conventional hydraulic fracturing operations [7,8]. These polymer-based fluids leave residues in the immediate fracture area of the rock matrix, impairing the formation and lowering pore conductivity. Near-fracture damage is a complex mechanism that can be characterized and studied as one of the components of the completion pseudo-skin factor [9]. Bisweshwar Gosh et al. (2020) [10] reported the problem of formation damage in the fracturing-treated zones caused by polymeric residuals resulting in fracturing water entrapments. Only 30% to 45% of the injected guar-based polymer fluids returns from the well during the flow-back period. Surfactant-based viscoelastic (SBVE) or viscoelastic surfactant (VES) fluids have emerged as a solution to the above issues and an alternative to polymeric fluids for hydraulic fracturing operations. The wormlike micelles (WLMs) of surfactant form smart self-organizing structures that are responsible for the viscoelastic properties of the fluid [11,12,13,14,15,16,17,18] (see Figure 1).

SBVE fluids are polymer-free fluids containing entangled wormlike micelles (WLM) which provide viscosity high enough to carry the proppant and sufficient elasticity to prevent fluid degradation during non-Newtonian fluid flow in the wellbore. Great interest has been raised in studying these colloidal systems of WLMs due to their polymer-like behavior combined with their high influenceability, which is useful in many industries [16]. Most importantly, the living nature of the cylindrical micelles, which are destroyed continuously and recombined, provides them with tremendous advantages over similar polymer systems; SBVE/VES gels have high sensitivity to minor change, and have the ability to fully restore their properties [17].

The micellization process accelerates in the presence of a suitable counterion reagent [15,19,20,21,22,23]. The enthalpy of micellization and Gibbs free energy for micellization seems to be the lowest for NO_3_^-^ compared to other inorganic anions [20], which indirectly indicates the entropy of micellization in CTAB solution. K. Kuperkar et al. [19] investigated viscoelastic solutions of wormlike micelles formed in the aqueous cationic surfactant CTAB in the presence of the salt reagent NaNO_3_. Adding NaNO_3_ to CTAB micelles led to a decrease in the surface charge of the ellipsoidal micelles and a resulting increase in their length. 

**Figure 2:**
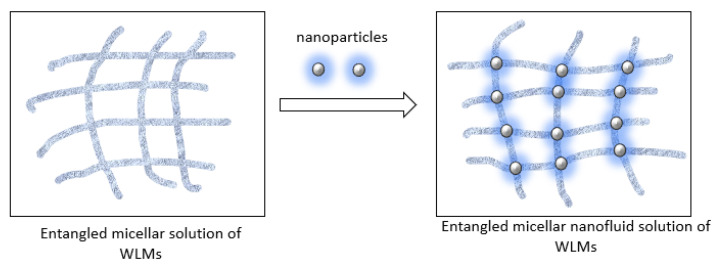
Formation of SBVE nanofluid system of entangled micellar solution of WLMs and NPs.

These viscoelastic systems are reported to show more improved rheological properties and stable structures in the presence of metal oxides or other suitable nanoparticles (NPs) [24,25,26,27,28,29,30,31,32,33]. Bandyopadhyay et al., Fan et al., and Luo et al. explained the indirect effect of nanoparticles on the growth of micelles by promoting electrostatic shielding between micelles acting as macroions without any interaction between the micelles [34,35,36]. The nanoparticles support crosslinking of the entangled micellar surfactant system by joining the endcaps and intersection points of cylindrical micelles, which provides stable rheological properties under adverse environmental conditions (see Figure 2).

**Figure 3:**
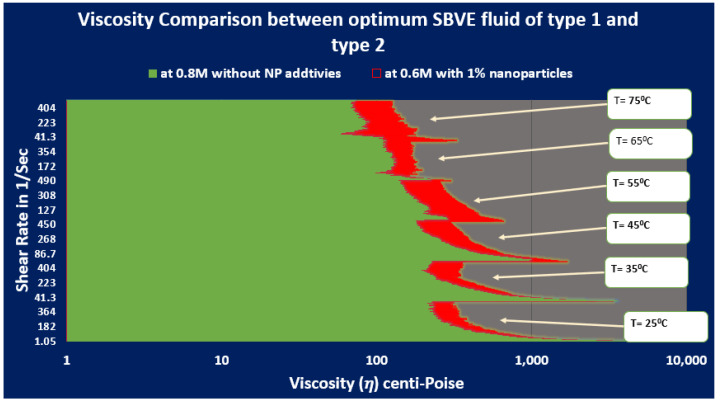
Rheology comparison in terms of viscosity of the optimum viscoelastic fluids of type 1 (without NP additives) and type 2 (with 1 wt% of ZnO NP additives) [37].

Recently, we have reported that ZnO nanoparticle dispersion could improve the rheology of the viscoelastic fluid system of cationic cetyltrimethylammonium bromide (CTAB)-based fluids (see Figure 3) [37]. This research article is a continuation of part of the same research. Previously, we reported the effect of ZnO NPs on the rheology of viscoelastic fluids of 0.1 M CTAB concentration and that the optimum surfactant-based viscoelastic (SBVE) nanofluid (type 2 fluid with ZnO NPs additives) showed better rheology than optimum SBVE fluid (type 1 fluid without NPs additives).

In this study, we perform a comparative analysis of SBVE fluids and polymeric guar gum gel fluid to investigate the potential of the former for on-field application during hydraulic fracturing operations. We ascertain that the previous optimum SBVE fluids of type 1 and type 2 show poor rheology compared to guar gum gel fluid. Therefore, a new SBVE fluid system with high surfactant concentration (0.2 M CTAB) both with and without 1 wt% ZnO NPs additives and varying counter-ion salt reagents was prepared and investigated in this study. Those fluids with high surfactant concentrations are categorized as type 3 fluids and type 4 nanofluids. Table 1 describes all the fluids prepared and categorized in this study as type 1, type 2, type 3, and type 4 subcategories. The rheology was analyzed using a rotational rheometer with varying shear rates from 0.1 to 500 s^−1^ at 5 s^−1^ intervals and different temperatures of all SBVE fluids, nanofluids, and guar gum gel fluid. The fluid concentration was optimized based on the rheological behavior of the fluids when compared with fluids in the same category at 25 °C. It is important to study the factors affecting the key controlling properties of the fluid viscosity and flow to ensure its optimum application during fracturing operation. These are the shear rate due to the non-Newtonian nature of the fluid and the temperature of the surroundings [3,4,37,38,39,40]. The prepared fracturing fluid flows through the high-pressure surface facilities to the well, then to the bottom of the well towards targeted subsurface reservoir rock during fracturing operations. Therefore, the rheological analysis performed at different temperature conditions varies from standard room temperature 25 °C to high temperature up to 75 °C considering the flow of fluid through the surface fracturing facilities, well, and reservoir rock. The non-Newtonian property of the fluid, which affects its viscosity during flow, is its shear rate conditions. The rheology is studied for wide range of shear rates up to 500 s^−1^. The rheology of the optimum fluids and nanofluids compared to guar gum gel fluid at varying shear rates (from 0.1 to 500 s^−1^) and under different temperature conditions of 25 °C, 35 °C, 45 °C, 55 °C, 65 °C, and 75 °C. Therefore, the rheology of the fluids is studied and compared on 100 different shear rate conditions under room temperature for optimization of fluid in each subcategory and the optimum fluids are compared with each other and guar gum under all six different temperature conditions. The viscosity data were plotted and compared using clustered bar charts to best visualize all actual viscosity data on all 600 points of different temperature and shear rate conditions.

## 2. Materials and Methods

**Figure 4:**
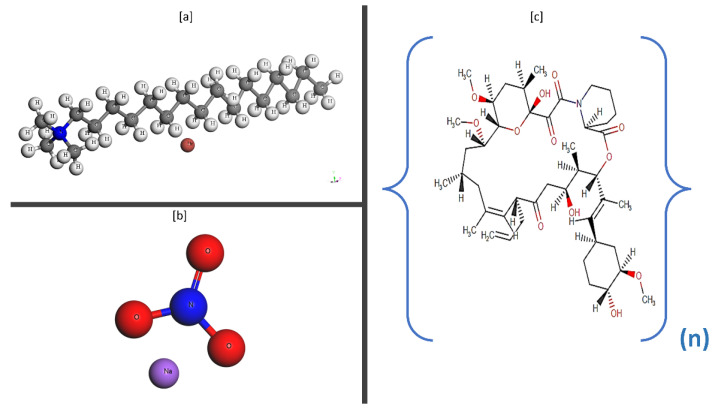
Molecular structure of (**a**) Cetyltrimethylammonium ammonium bromide, (**b**) Sodium Nitrate, and (**c**) Guar Gum.

Cetyltrimethylammonium bromide (CTAB), or cetrimonium bromide, is a cationic quaternary ammonium surfactant. The chemical formula for CTAB is ([(C_16_H_33_)N(CH_3_)_3_]Br), and the molar mass is 364.447 gm/mol. Its molecular structure is illustrated in Figure 4. Cationic surfactant cetyltrimethylammonium bromide (98% pure) from Loba Chemie Pvt. Ltd. was obtained from Sigma Aldrich (Mumbai, India). AR grade Sodium Nitrate salt reagent (with a minimum assay of 99%) was obtained from ACS chemicals (Mumbai, India). The molar mass was 84.994 gm/mole, and the molecular structure is shown in Figure 4. The nano-dispersion of ZnO NPs (<100 nm particle size TEM), 20 wt% in H_2_O, was obtained from Sigma Aldrich. The guar gum was obtained from Swastik gum industries (Ahmedabad, India).

### 2.1. Preparation of Type 3 Fluids without Nanoparticle Additives

**Figure 5:**
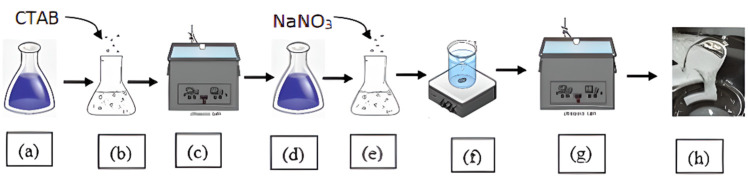
Process of preparing type 1 viscoelastic fluid without nanoparticle additives. Steps (**a**–**c**) show the process for the preparation of surfactant solution and steps (**d**–**h**) depict the process of SBVEF formation; steps (**c**,**g**) show ultrasonication processes and step (**f**) shows magnetic stirring [37].

The type 3 fluids were prepared similarly to the type 1 fluid prepared in previous study [37] with different surfactant and salt concentrations. Figure 5 illustrates the method of preparing fluids in the type 1 and type 3 categories.

### 2.2. Preparation of Type 4 Fluid with Nanoparticle Additives

**Figure 6:**
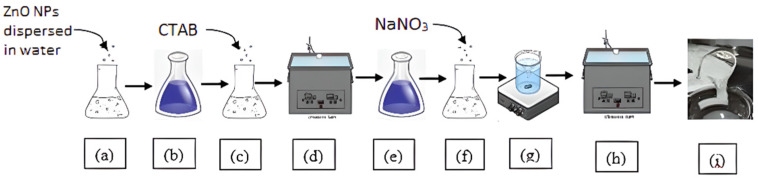
Process of preparing type 2 viscoelastic fluid with nanoparticle additives. Steps (**a**–**d**) show the process for the preparation of surfactant nanofluid solution and steps (**e**–**i**) depict the process of formation of SBVEF; steps (**d**,**h**) show ultrasonication process and step (**g**) shows magnetic stirring [37].

The type 4 fluids were prepared similarly to the type 2 fluids prepared in the previous study [37], with 1 wt% of ZnO NPs dispersions and different surfactant and salt concentrations. Figure 6 illustrates the method used to prepare the SBVE nanofluids in the type 2 and type 4 categories.

### 2.3. Preparation of Guar Gum Fluid Gel

**Figure 7:**
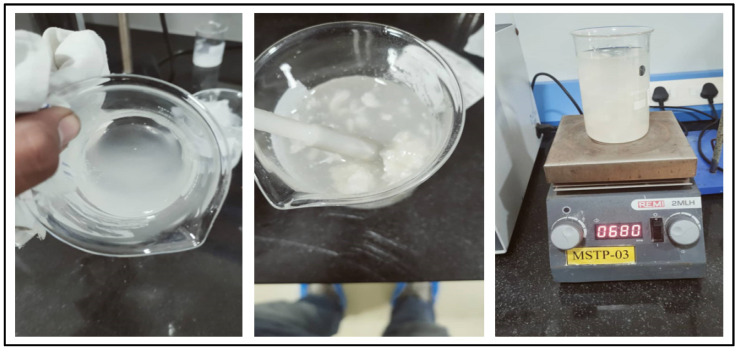
Guar gum gel fluid with (Middle) and without (Left) agglomerates, showing magnetic stirring process (Right).

Preparing guar gum gel fluid was not easy, as it makes agglomerates. The guar powder was mixed carefully with continuous magnetic stirring of heated water to avoid agglomeration problems. Figure 7 shows the method of preparing guar gum gel fluid and formation of clusters or agglomerates of guar gum powder when it was prepared at a concentration higher than 1 wt% or when the experiment was conducted without precautions or continuous agitation.

## 3. Rheometric Analysis and Observations

**Figure 8:**
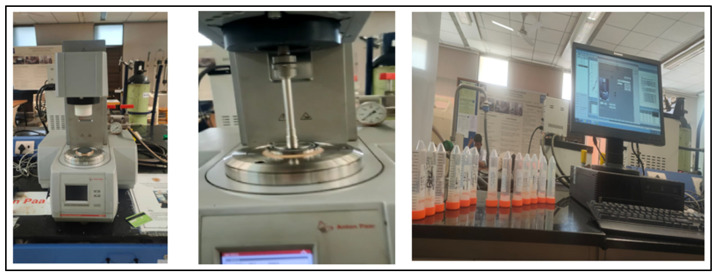
Anton-par rheometer model MCR52 (at PE laboratory, PDEU).

The present research investigated whether the prepared SBVE fluids/nanofluids have comparative rheological characteristics to guar gum gel fluid. The rheometric analysis of the SBVE fluids/nanofluids and guar gum fluid was performed using an Anton- Par rotational rheometer (MCR2 Model). The Parallel Plate geometry (plate diameter: 40 mm) was implemented at a gap of 0.17 mm for all the fluid analyses (see Figure 8). The viscosity is a key parameter that influences and controls flow behavior in pipeline hydraulics [41]. The proppant carrying capacity of the fluid and potential to form fractures during hydraulic fracturing are mainly controlled by the viscosity of the fracturing fluid [10,42,43,44]. 

The viscosity was observed by varying the shear rate from 0.1 to 500 s^−1^ at a difference of 5 s^−1^ under different temperature conditions of 25 °C to 75 °C by 10 °C intervals. Therefore, the viscosity data of the fluids was studied and compared for 100 different shear rate conditions under room temperature for optimization of the fluids in the type 1, type 2, type 3 and type 4 categories. The optimum fluids were compared with each other and with guar gum under all different temperature and shear rate conditions. The viscosity data were plotted and compared on clustered bar charts to visualize the differences in terms of the actual viscosity data of the different fluids on all 600 points for a wide range of shear rate and temperature conditions.

### 3.1. Rheometric Analysis of Guar Gum Gel Fluid 

**Figure 9:**
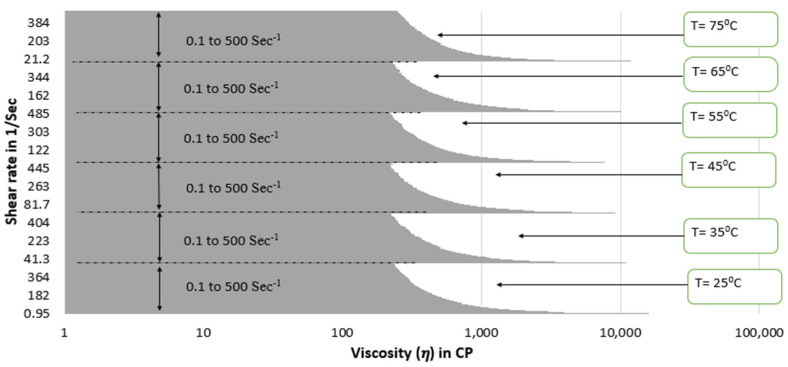
Viscosity plot of 1 wt% guar gum gel fluid at different temperatures and shear rate ranges.

Figure 9 illustrates the viscosity of guar gum gel at varying shear rate conditions (From 0.1 to 500 s^−1^) and at different temperatures of 25 °C, 35 °C, 45 °C, 55 °C, 65 °C, and 75 °C.

### 3.2. Rheometric Analysis of Type 3 Fluids and Optimization of Fluid Concentration

The viscosity plots show different values with the change in salt concentration. The values increase for the fluids with increased salt concentration from 0.2 M to 0.4 M and slightly decreases at 0.6 M. Further, the values reflect an increasing trend as the salt concentration increases to 1.2 M. The viscosity plot of 1.2 M counter ion concentration fluid reflect higher viscosity values maintained under low and high shear rate conditions.

Further, when analyzing fluids in this category with increased salt concentration, the viscosity plot depicts reduced values for the fluid with 1.4 M NaNO_3_. Thus, the fluid with 1.2 M NaNO_3_ concentration is the optimum fluid of the type 3 category of high surfactant concentration (0.2 M CTAB) fluids. However, a concentration of 1.0 M provides similar values under certain exceptional shear rate conditions.

**Figure 10:**
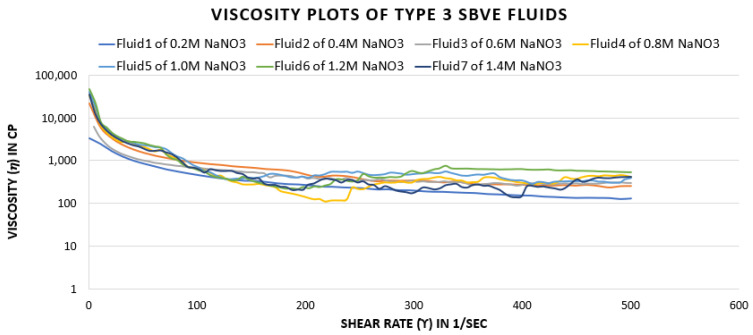
Viscosity curves of SBVE fluids of type 3 category with varying NaNO_3_ concentration at 25 °C.

**Figure 11:**
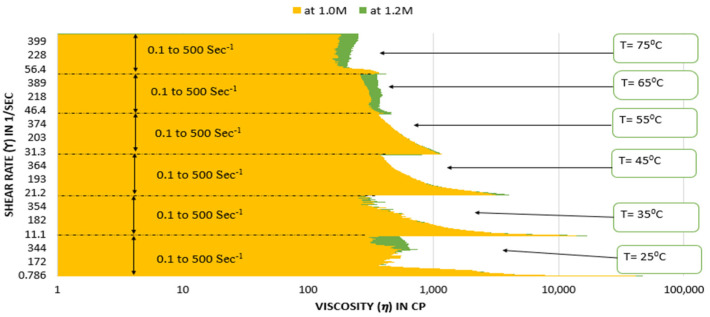
Comparison of viscosity plot of type 3 viscoelastic fluids containing 1.0 M and 1.2 M NaNO_3_ concentration at different temperatures.

Figure 10 and Figure 11 depict that the fluid with 1.2 M salt concentration seems to have high viscosity in almost all temperature and shear rate conditions. Therefore, it can be concluded that the fluid with 1.2 M salt concentration is the optimal fluid in the type 3 category.

### 3.3. Rheometric Analysis of Type 4 Fluids and Optimization of Concentration

In this case, the viscosity behaviour seems to be different from other cases, as at 0.2 M salt concentration the plot resembles very smooth viscosity data. The values decrease with increasing shear rate without any ups or downs.

**Figure 12:**
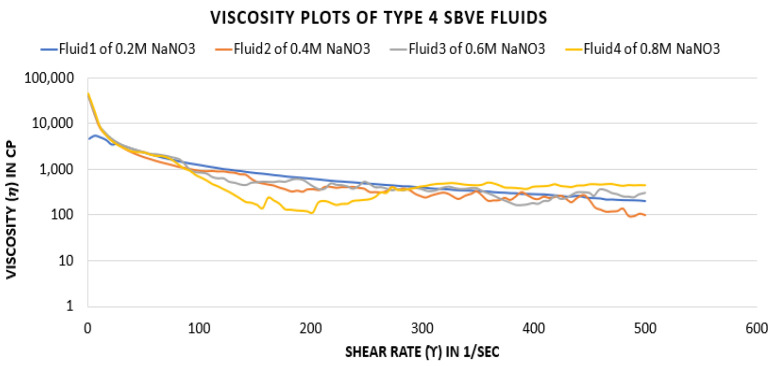
Viscosity curves for SBVE fluids of type 4 category with varying NaNO_3_ concentration at 25 °C.

At 0.4 M concentration, the viscosity data seem to be higher at starting shear rate values below 100 s^−1^. Then, the viscosities provide similar values to those obtained with a shear rate of 450 (See Figure 12).

Further, lesser viscosity values are depicted at a high shear rate after 450 s^−1^ compared to the 0.2 M concentration. The plots of 0.6 M and 0.8 M show higher values at the starting shear rates. However, at 0.8 M the viscosity decreases for shear rates of 100 to 200, then the values increase. At the same time, the 0.6 M plot seems similar to the 0.2 M plot, with only a few fluctuations in values. The 0.4 M concentration fluid shows less value at 25 °C, while at the higher temperature the 0.4 M fluid shows higher viscosity values. Thus, it can be concluded that the 0.4 M sodium nitrate concentration fluid is optimal among this group of fluids.

### 3.4. Comparative Analysis of Optimal SBVE Fluids to Guar Gum Gel Fluid

Initially, the rheology of the viscosity plots of guar gum gel fluid compared with the optimum SBVE fluid (without nano-additives) and nanofluid (with 1 wt%ZnO NPs additives) in the previous study were type 1 and type 2 SBVE fluids. The fluids had a CTAB concentration of 0.1 M, and the optimal counter-ion NaNO_3_ concentrations were 0.8 M and 0.6 M, respectively.

**Figure 13:**
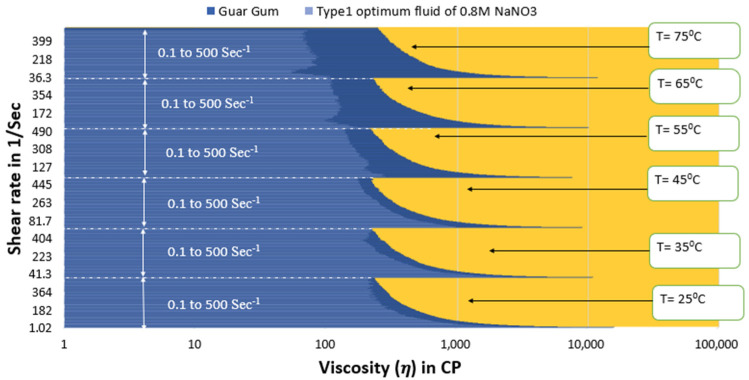
Viscosity plot comparison between guar gum and type 1 optimum SBVE fluid.

**Figure 14:**
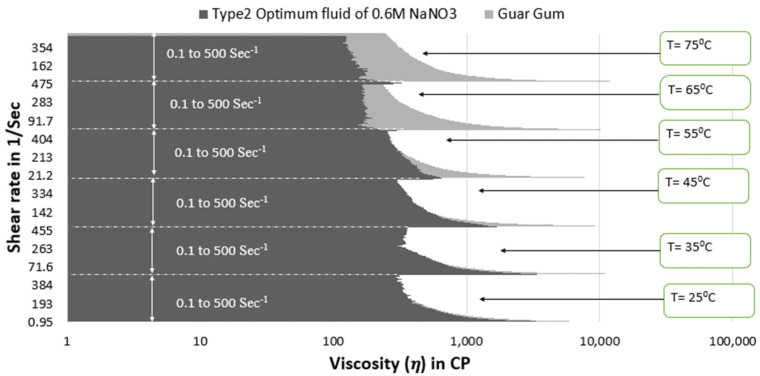
Viscosity plot comparison between guar gum and type 2 optimum SBVE fluid.

Figure 13 and Figure 14 show that the viscosity of guar gum is better than type 1 optimum fluid under all temperature and shear rate conditions. However, the viscosity of type 2 optimum fluid remains similar to guar gum until 55 °C, then the type 2 optimum SBVE nanofluid show lesser viscosity values at 65 °C and 75 °C for all shear rate conditions.

It can be concluded that the type 1 and type 2 optimum fluids cannot replace guar gum, as they show poor rheology under elevated shear rate and temperature (ESRT) conditions. Therefore, it was necessary to develop more fluids with improved rheology. We prepared and optimized type 3 and type 4 fluids with increased surfactant concentration under the hypothesis that they would have comparable rheology to guar gum gel fluid.

**Figure 15:**
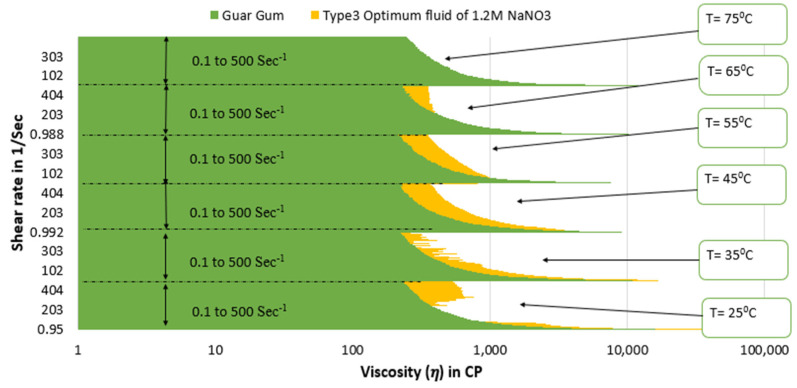
Viscosity plot comparison between guar gum and type 3 optimum SBVE fluid.

**Figure 16:**
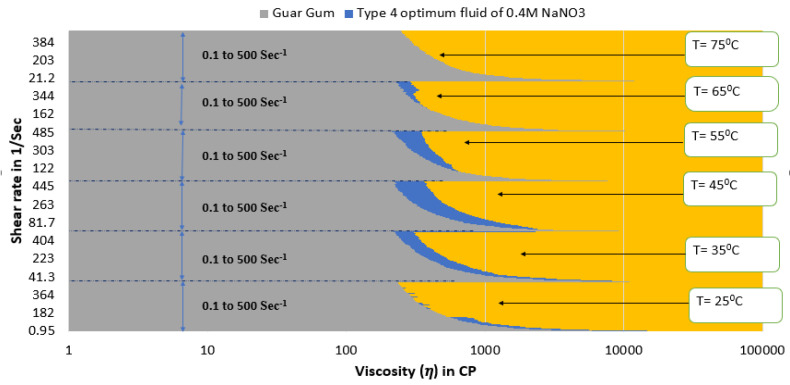
Viscosity plot comparison of guar gum and type 4 optimum SBVE nanofluid.

Figure 15 and Figure 16 depict a comparison of the estimated viscosities of guar gum gel fluid with type 3 and type 4 SBVE optimum fluid and nanofluid under different temperature conditions for a range of shear rates up to 500 s^−1^. The figures reflect that the optimum fluid and nanofluid show better viscosities than guar gum gel fluid up to 55 °C under all conditions. The guar gum shows similar viscosity values under initial shear rate conditions (up to 150 s^−1^ shear rate conditions) at 65 °C for both optimum SBVE fluid and nanofluid. In contrast, the optimum fluid and nanofluid show better viscosity at high shear rate conditions. Further, the guar gum fluid shows better viscosities at 75 °C temperature when compared to both optimum fluid and nanofluid at low shear rate conditions. The optimum type 4 nanofluid shows poor rheology compared to guar gum fluid at high shear rate conditions. However, the type 3 optimum fluid shows similar viscosities to guar gum at high shear rate conditions (see Figure 15) at 75 °C.

Therefore, type 3 optimum fluid can be considered a potential alternative candidate with comparable rheological characteristics to polymeric guar gum fluids.

### 3.5. Comparative Analysis of All Optimum Fluids to Finding the Overall Optimum Fluid 

**Figure 17:**
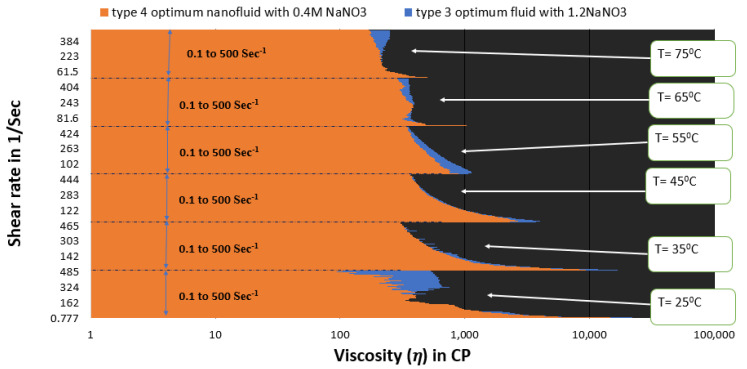
Viscosity plot comparison between optimum SBVE fluids of types 3 and 4.

The prepared fluids of type 3 and type 4 belong to the group of high surfactant concentration fluids. These optimum fluids were compared to the type 3 and 4 categories (Figure 17). The type 3 optimum fluid shows better viscosity for the entire shear rate range, even under high temperature conditions. Apparently, ZnO NPs do not benefit the entangled micellar structure of high surfactant concentration SBVE fluids (types 3 and 4) prepared in this study.

Optimum nanofluid of the type 2 category showed better rheology than other fluids with low surfactant concentration (0.1 M CTAB) [37]. Therefore, the rheology of the best low-surfactant fluid (type 2 optimum nanofluid) was compared with the high-surfactant fluid, which is the type 3 optimum fluid.

**Figure 18:**
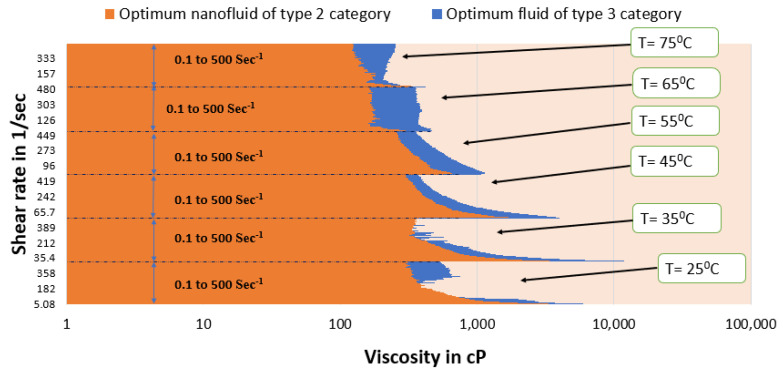
Comparison of optimum SBVE fluids of types 3 and 2.

Figure 18 shows that the type 3 optimum fluid has better rheology than the optimum type 2 fluid under almost all shear rate and temperature conditions. Therefore, it can be concluded that the type 3 optimum fluid is best out of all other fluids prepared in this study. In other words, the synthesized SBVE fluid of 0.2 M cetyltrimethylammonium bromide and 1.2 M sodium nitrate is the overall optimum fluid. 

Statistical parameters are compared of viscosity data produced by optimum fluid (type 3) and nanofluid (type 4), and guar gum gel fluid, such as the standard deviation, mean, and standard error of the mean. The viscosity data of all three fluids was analyzed repeatedly at 100 same shear rate conditions under six different temperatures.

The optimum SBVE fluid shows the highest mean viscosity value compared to the optimum SBVE nanofluid and guar gum. The standard deviation is higher for the optimum fluid than for the other fluids, meaning that the viscosity values are more dispersed for the optimum SBVE fluid around its mean value of 818.09 centipoise. In contrast, guar gum shows the lowest SD value. Therefore, the viscosity values of guar gum are clustered around its mean value of 688 centipoise (see Table 2). The standard deviation (SD) measures how dispersed the data are around the mean. Lower values of SD mean that data are clustered around the mean, while high SD values indicate that the data are more spread out. The viscosity of the optimum SBVE fluid and nanofluid show dispersed viscosity values, while guar gum shows comparatively clustered viscosity values around the mean viscosity of 1258.263 cp. The standard error of the mean (SEM) signifies how different the population means are likely to be from a sample mean. It indicates how much the sample mean would vary if repeating the study using new samples from within a single population. SEM values are higher in case of the optimum SVBE fluid and nanofluid than for the guar gum gel fluid due to the high influence of temperature on the viscosity of SBVE fluids compared to on the guar gum fluid during repetitive rheology analysis at the same shear rate range and under six different temperature conditions.

## 4. Discussion

In our previous paper, we proposed ZnO nanoparticle-enhanced nonpolymeric SBVE fluids for hydraulic fracturing applications. However, the fluids were not compared with any polymeric fluids applied conventionally in fracturing operations. In this study, the rheology of the fluids was compared with traditional guar gum gel fluids to develop and optimize new high-surfactant SBVE fluids based on the hypothesis of obtaining fluids with highly improved rheology. The key findings of this study are as followings:

- The low-surfactant optimum SBVE fluids of types 1 and 2 cannot replace guar gum, showing poor rheology under elevated shear rate and temperature (ESRT) conditions.
- The high-surfactant SBVE fluids (type 3) and nanofluids (type 4) of 0.2 M CTAB concentration show optimal rheology when prepared at 1.2 M and 0.4 M counter-ion sodium concentration, respectively.
- The optimal type 3 fluid and type 4 nanofluid show better viscosity than guar gum gel fluid up to 55 °C under all conditions.
- The guar gum fluid shows similar viscosity values under initial shear rate conditions (up to 150 s^−1^ shear rate conditions) at 65 °C to both optimum SBVE fluid and nanofluid. However, the optimum fluid and nanofluid show better viscosity under high shear rate conditions.
- The guar gum fluid shows better viscosities at 75 °C temperature when compared to both the optimum fluid and nanofluid under low shear rate conditions.
- The optimum type 4 nanofluid shows poorer rheology than guar gum fluid under high shear rate conditions; however, the type 3 optimum fluid shows similar viscosities to guar gum at high shear rate conditions at 75 °C.
- The type 3 optimum fluid shows better viscosity for the entire shear rate range, even under high-temperature conditions, than optimum fluids of type 1, type 2, and type 4. Therefore, the synthesized SBVE fluid of 0.2 M cetyltrimethylammonium bromide and 1.2 M sodium nitrate is the best out of all fluids prepared in this study. In other words, it is the overall optimum fluid.
- As reported previously, ZnO NPs additives can improve the rheological characteristics of SBVE fluids of low surfactant concentration. However, this part of the study of high surfactant SBVE fluids indicates that SBVE optimum fluid in the category with no nano-additives (type 3) shows better rheology than the optimum nanofluid prepared with the addition of 1 wt% ZnO nano dispersion (type 4).

**Figure 19:**
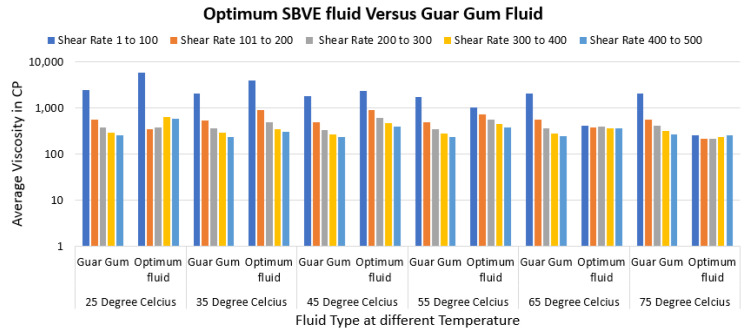
Average viscosity comparison of the rheology of the overall optimum fluid and guar gum.

Moreover, the type 3 optimum fluid seems to have comparable rheology to guar gum fluid under all conditions. For field implementation, the average viscosity values at shear rate groups of 1 to 100, 100 to 200, 200 to 300, 300 to 400, and 400 to 500 s^−1^ can be compared (Figure 19). The overall optimal fluid and the guar gum show similar average viscosity under all ESRT conditions.

Therefore, the presented novel optimal surfactant-based viscoelastic fluid of the type 3 category has the potential to replace polymeric guar gum fluid and can be implemented as a novel nonpolymeric fracturing fluid composition during hydraulic fracturing operations. 

## Figures and Tables

**Table 1:** SBVE fluids and nanofluids prepared in this study.

Group 1 Fluids (Low Surfactant Concentration Fluids)		Group 2 Fluids (High Surfactant Concentration Fluids)	
Type 1 Fluids	Type 2 Nanofluids	Type 3 Fluids	Type 4 Nanofluids
Fluid 1 (0.2 M NaNO_3_)	Fluid 1 (0.2 M NaNO_3_)	Fluid 1 (0.2 M NaNO_3_)	Fluid 1 (0.2 M NaNO_3_)
Fluid 2 (0.4 M NaNO_3_)	Fluid 2 (0.4 M NaNO_3_)	Fluid 2 (0.4 M NaNO_3_)	Fluid 2 (0.4 M NaNO_3_)
Fluid 3 (0.6 M NaNO_3_)	Fluid 3 (0.6 M NaNO_3_)	Fluid 3 (0.6 M NaNO_3_)	Fluid 3 (0.6 M NaNO_3_)
Fluid 4 (0.8 M NaNO_3_)	Fluid 4 (0.8 M NaNO_3_)	Fluid 4 (0.8 M NaNO_3_)	Fluid 4 (0.8 M NaNO_3_)
Fluid 5 (1.0 M NaNO_3_)	Fluid 5 (1.0 M NaNO_3_)	Fluid 5 (1.0 M NaNO_3_)	
Fluid 6 (1.5 M NaNO_3_)	Fluid 6 (1.5 M NaNO_3_)	Fluid 6 (1.2 M NaNO_3_)	
Fluid 7 (2.0 M NaNO_3_)	Fluid 7 (2.0 M NaNO_3_)	Fluid 7 (1.4 M NaNO_3_)	

**Table 2:** Statistical parameters and viscosity data of optimum SBVE fluid and nanofluid of type 3 and type 4.

Fluid	SD	Mean	SEM
Optimum SBVE nanofluid of type 4	1892.556	689.6557	77.26326
Optimum SBVE fluid of type 3	2384.984	818.0965	97.44782
Guar Gum gel	1258.263	688	51.36839

## Data Availability

Not applicable.
